# Abscission Is Regulated by the ESCRT-III Protein Shrub in *Drosophila* Germline Stem Cells

**DOI:** 10.1371/journal.pgen.1004653

**Published:** 2015-02-03

**Authors:** Neuza Reis Matias, Juliette Mathieu, Jean-René Huynh

**Affiliations:** 1 Department of Genetics and Developmental Biology, Institut Curie, Paris, France; 2 CNRS UMR3215, Inserm U934, Paris, France; The University of North Carolina at Chapel Hill, UNITED STATES

## Abstract

Abscission is the final event of cytokinesis that leads to the physical separation of the two daughter cells. Recent technical advances have allowed a better understanding of the cellular and molecular events leading to abscission in isolated yeast or mammalian cells. However, how abscission is regulated in different cell types or in a developing organism remains poorly understood. Here, we characterized the function of the ESCRT-III protein Shrub during cytokinesis in germ cells undergoing a series of complete and incomplete divisions. We found that Shrub is required for complete abscission, and that levels of Shrub are critical for proper timing of abscission. Loss or gain of Shrub delays abscission in germline stem cells (GSCs), and leads to the formation of stem-cysts, where daughter cells share the same cytoplasm as the mother stem cell and cannot differentiate. In addition, our results indicate a negative regulation of Shrub by the Aurora B kinase during GSC abscission. Finally, we found that Lethal giant discs (lgd), known to be required for Shrub function in the endosomal pathway, also regulates the duration of abscission in GSCs.

## Author Summary

Abscission is the final step of cytokinesis which allows the physical separation of sister cells through the scission of a thin cytoplasmic bridge that links them at the end of mitosis. The duration of abscission varies depending on cell types, indicating that the event is developmentally regulated. Recently, we have identified two kinases, Aurora B and CycB/Cdk-1, which regulate the timing of abscission in germ cells and in mammalian cells. However, these kinases are upstream regulators and do not perform abscission per se. Here, we show that Shrub, a potential target of Aurora B and one of the most downstream effectors of abscission, is required for complete abscission in germline stem cells. In the absence of Shrub, the mother stem cell remains linked to its daughter cells, which then share the same cytoplasm and cannot differentiate. Loss of Shrub and Aurora B have opposite effects on abscission duration suggesting that Aurora B regulates negatively Shrub. We further show that Shrub acts together with its interactor Lethal giant disc to ensure proper abscission timing.

## Introduction

Abscission is the last step of cytokinesis when sister cells linked by a thin cytoplasmic bridge become physically separated. It takes place on the side of an electron dense structure called the midbody that resides within the bridge. Unexplored for many years, this late step of cell division has begun to be characterized at the cellular and molecular level in the last decade as a result of recent advances in microscopy and genetic engineering [1]. Our understanding of abscission originates from studies carried out mainly in yeast and in mammalian cells in culture. However, features like the duration of abscission vary greatly from one cell type to another. It lasts a few hours in mammalian cells, while in sea urchin embryos, the completion of cell division only occurs during the S phase of the next cycle [2]. Abscission is completely blocked in germ cells of most species at some point during normal development [3]. How abscission timing is regulated in a developmental context remains however poorly characterized.

During abscission, membrane scission happens at a secondary ingression point in the bridge that appears just before the cut [4,5,6]. At this site, microtubules overlapping in the bridge are severed by the AAA ATPase Spastin, and actin filaments are cleared by modifications of the lipid content of the membrane mediated by the PIP2-phosphatase OCRL [7,8]. A secondary constriction is thought to be formed and then abscised, by a set of proteins belonging to the Endosomal Sorting Complex Required for Transport-III (ESCRT-III) machinery and the vacuolar protein sorting 4 (VPS4). The subunits of the ESCRT-III complex, including CHMP4B, and the most downstream component VPS4 are relocated at the exact site of the cut just before abscission occurs [5,6]. The ESCRTs have the ability to self-assemble into spiral filaments, a structure that has been described beside the midbody that would allow membrane curvature and scission [1,9]. The timing of abscission depends on the local recruitment of the ESCRT-III machinery. This can only occur after mitotic exit when PLK1 gets degraded, and thereby allows the centrosomal protein 55 (CEP55) to localize to the midbody [10]. This in turn permits the sequential recruitment of the ESCRT-I component TSG101 and ALIX, and finally the ESCRT-III machinery [11,12,13]. Although recruited by the ESCRT-I complex during Multi Vesicular Bodies (MVBs) formation, the ESCRT-II complex does not appear to be involved in abscission in mammalian cells. In mouse males spermatocytes, the binding of CEP55 to ALIX and TSG101 is inhibited, therefore abscission does not occur and a stable bridge is formed [14]. Abscission can also be blocked or delayed by the presence of lagging strands of DNA in the cytoplasmic bridge between two sister cells. Elegant work identified this checkpoint in yeast and mammalian cells, and demonstrated that it delays abscission until the lagging DNA bridges are resolved. It has thus been named the NoCut checkpoint. An important molecular player of this checkpoint is the essential mitotic kinase Aurora B [15,16]. Aurora B delays abscission by phosphorylating a member of the ESCRT-III complex, CHMP4C, a close paralog of the filament forming CHMP4B required for abscission [17,18]. Recent work suggests that this delay may be mediated by the retention at the midbody ring of the terminal effector of abscission Vps4 by CHMP4C and ANCHR proteins [19]. The temporal control of abscission is thus highly regulated by a complex molecular machinery that is still not fully understood. In addition, whether the conserved ESCRT machinery is regulating abscission in *Drosophila* has not yet been explored.

**Figure 1:**
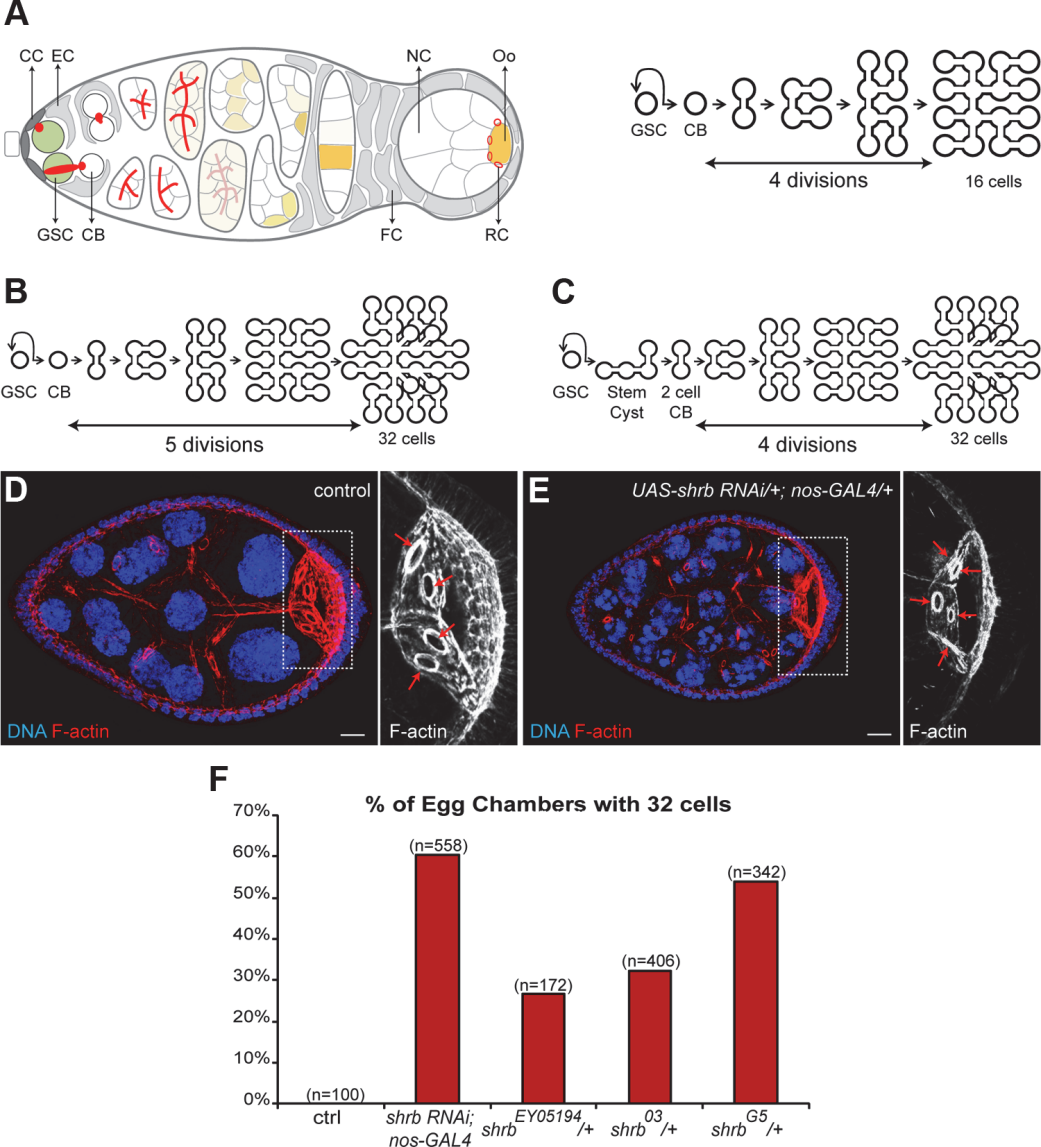
Loss of the ESCRT-III subunit Shrub in germline cells induces the formation of egg chambers with 32 cells. (A) Schemes of a germarium (left) and germline divisions (right). At its anterior tip, the germline stem cell (GSC, green) divides asymmetrically and produces a cystoblast (CB). Cap cells (CC) maintain GSC stemness, while escort cells (EC) promote CB differentiation. The CB goes through 4 divisions forming a cyst of 16 cells, 15 nurse cells (NC) and 1 oocyte (Oo, yellow). The cyst is encapsulated by follicular cells (FC) and buds out of the germarium. The abscission of the GSC/CB is complete, while in the following 4 divisions it is incomplete. The oocyte shares the cytoplasm with the NC through 4 ring canals (RC). The spectrosome in the GSC and the fusome in its progeny (red, left scheme) are germline-specific organelles. Anterior is on the left, posterior on the right. (B and C) Schemes representing 2 possible ways of explaining the 32-cell cysts. (B) A fifth mitosis. (C) A delay in GSC abscission. (D and E) Stage 7 egg chambers from WT or *UAS-shrb RNAi/+; nos-GAL4/+* stained with DAPI (DNA, blue) and phalloidin (F-actin, red). On the right, close-up on oocytes. Red arrows indicate the four ring canals in the control oocyte and the five ring canals in the mutant background. (F) Fraction of egg chambers exhibiting 32 cells on the y axis. Genotypes are on the x axis. Scale bar: 10 μm.

Recently, we have used the *Drosophila* female germline to study abscission in a developmental context and in a genetically amenable system [20]. *Drosophila* germ cells regulate abscission differentially according to the developmental stage. Germline stem cells (GSCs) are located at the very anterior of region 1 of the germarium, in contact with somatic cells called cap cells and escort cells, which regulate their behavior[21]. Each stem cell divides asymmetrically to generate one stem cell, which stays in contact with cap cells in the niche, and a second daughter cell positioned outside of the niche. The daughter cell starts to transcribe the gene *bam*, which is necessary and sufficient to trigger the differentiation of the daughter cystoblast (CB). This differentiation is characterized by four rounds of synchronous and incomplete divisions, giving rise to a cyst of 16 cells made of 15 nurse cells and one oocyte. In the resulting cyst, each cytokinesis is arrested and all sister cells share the same cytoplasm through ring canals. In contrast, cytokinesis between the GSC and the CB is complete. It is, however, very slow and GSCs and CBs remain linked at least until the following S-phase. The orientation and synchrony of these divisions are controlled by a germline-specific organelle, called the fusome, which is made of ER-derived vesicles (Fig. 1A)[21]. The fusome is partly inherited from the round spectrosome of the GSCs (also made of ER-derived vesicles), and partly newly formed at the midbody during each division. Fusion between fusome precursors creates a continuum of vesicles penetrating each canal and connecting all the cells within a cyst. The fusome thus appears branched in dividing germline cysts. The fusome starts to degenerate and disappears when the germline cyst enters the meiotic zone or region 2 of the germarium [22,23].

In a genetic screen, we isolated the first mutations in *aurora B* and *survivin*, a regulatory subunit of the Chromosomal Passenger Complex (CPC) in *Drosophila* [20,24]. Using allelic series of gain- and loss-of-function of these genes, we demonstrated that the function of Aurora B as an abscission timer is conserved during the development of germline cells. Enhancing Aurora B activity delays abscission in GSCs and multiple divisions can occur before the preceding abscissions are completed. This leads to the formation of stem-cysts, a structure composed of several cells with stem cell-like properties still linket to the anterior GSC. In contrast, reducing Aurora B activity induces precocious abscission in GSCs and complete abscission in 2-cell cysts. A simple readout of these events is the number of germ cells per cyst. 32 cells or more per cyst are found when abscission is delayed in cyst, while 8 cells or less are observed in the case of a precocious abscission in the cyst [20]. In this study, we have analyzed the consequences of the loss of function of Shrub (Shrb), the single *Drosophila* homolog of CHMP4, on the development of the germline lineage. We found that Shrb was positively required for abscission, as mammalian CHMP4B; and it negatively regulated by Aurora B. In addition, we showed that Lethal giant discs (Lgd), which is known to be required for Shrb function in the endosomal pathway, was also involved in GSCs abscission regulation.

## Results

### 1. Loss of the ESCRT-III subunit Shrub (SNF7/CHMP4) in germline cells induces the formation of egg chambers with 32 cells

We performed a pilot RNAi screen for mutants affecting the number of germ cells per egg chamber. Mutations in *cyclin A, cyclin E* or their regulators affect the number of germ cells by modifying the number of divisions. High levels of Cyclin A or mutations in *encore* induce the formation of egg chambers with 32 cells by triggering a fifth mitosis in cysts (Fig. 1B) [25,26]. In contrast, mutations in *cyclinE* or *half-pint* give rise to egg chambers with 8 cells due to only 3 divisions [27,28]. Recently, we showed that changing the duration of abscission could alter the number of germ cells per egg chamber without modifying the number of cyst mitosis. We showed that delaying abscission in GSCs induces the formation of cystoblasts made of two cells instead of one, which results in the formation of egg chambers containing 32 cells after four divisions (Fig. 1C). In contrast, a faster abscission after the first cyst mitosis allows for the completion of cytokinesis in 2-cell cysts, and leads to the formation of two cysts of 8 cells after the remaining three divisions [20]. To find novel genes involved in these divisions, we used a collection of transgenic flies expressing shRNA designed to be efficient in germ cells (TRiP collection,[29]). We selected 230 transgenic lines targeting kinases, phosphatases and regulators of membrane trafficking, and expressed shRNAs specifically in germ cells using a *nanos*-GAL4 driver. Three females were dissected for each line and ovaries were stained with Hoechst to count the number of cells.

One line (HMS01767) induced a high percentage (61%, n = 558) of egg chambers containing 32 cells. In these mutant egg chambers, the oocyte was linked to nurse cells by 5 ring canals instead of 4 indicating that the extra germ cells were not the result of packaging defects (Fig. 1D-F). We also observed less penetrant phenotypes, such as binucleated cells, polyploid cells and a few egg chambers filled with tumor-like germ cells (S1 Fig.). The HMS01767 line encodes a short hairpin RNA directed against *shrub* (*shrb*). *shrb* encodes a subunit of the ESCRT-III complex and is the *Drosophila* homologue of *Snf7/CHMP4*. We tested the specificity of the RNAi line by using several *shrb* alleles: *shrb^G5^*, *shrb^O3^* and *shrb^EY05194^*. We could not recover any *shrb* homozygous mutant germ cells using the Flp/FRT technique. However, we noticed a high percentage of 32-cell egg chambers in flies heterozygous for these alleles: 27% (n = 172); 32% (n = 406) and 54% (n = 342) of the egg chambers has 32 cells in the *shrb^EY05194^/+, shrb^O3^/+ and shrb^G5^/+* females, respectively. We concluded that the RNAi was specific and that the gene dosage of *shrb* was important to regulate the number of germ cells per egg chamber.

### 2. The loss of Shrub in germline stem cells induces the formation of 32-cell cysts

**Figure 2:**
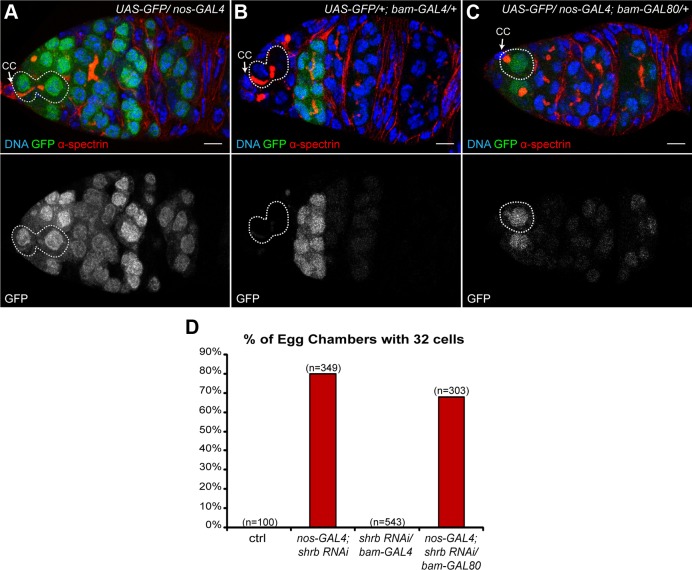
The loss of Shrub in germline stem cells induces the formation of 32-cell egg chambers. (A-C) Germaria expressing *UAS-GFP* under the control of different promoters and stained with DAPI (DNA, blue) and α-spectrin (fusome, red). (A) *nos-GAL4* is specific for germline cells; (B) *bam-GAL4* is specific for early differentiated cells in the cysts; (C) *nos-GAL4; bam-GAL80* is specific for germline, excluding the early cysts. Dotted lines surround GSC/CB pairs (A and B) and GSC (C). Cap cells (CC) are indicated. (D) Fraction of egg chambers exhibiting 32 cells on the y axis. Genotypes are on the x axis. Scale bar: 10 μm.

The cyst goes through four rounds of mitosis in a subpart of region 1 that can be identified by the expression of the gene *bam. bam* is weakly expressed in cystoblasts and 2-cell cysts, increases in 4-cell cysts, and peaks at 8-cell cysts before being switched-off quickly[30]. In mutants inducing one extra round of mitosis, the *bam*-expressing region is expanded posteriorly, indicating that the corresponding gene acts during the cyst divisions. In contrast, in abscission-defects mutant giving rise to 32-cell cysts, the genes are required in the GSCs before the cyst divisions. To distinguish between both possibilities, we expressed RNAi against *shrb* differentially in GSCs and dividing cysts. *nanos*-GAL4 drives expression in all germ cells of the germarium, whereas *bam*-GAL4 only in dividing cysts (Fig. 2A-B). We also expressed the same shRNA (HMS01767) against *shrb*, driven by *bam*-GAL4 to test if *shrb* was required in dividing cysts to control the number of divisions. In the later conditions, all egg chambers had 16 cells (0%, n = 543), in contrast to 80% (n = 349) of 32-cell cysts when driven by *nanos*-GAL4 (Fig. 2D). This result suggested that Shrb function is required in germ cells expressing *nanos*, but not *bam*, which are mainly the GSCs and a few pre-cystoblasts. To confirm this hypothesis, we combined *nanos*-GAL4 with a GAL80 repressor under the control of the *bam* promoter. In this background GAL80 repressed the activity of GAL4 in the *bam* expressing domain (Fig. 2C); as a consequence, *UAS-shrb-RNAi* was expressed only in GSCs and some pre-cystoblasts. In such ovaries, we found that 68% (n = 303) of egg chambers had 32 germ cells. We concluded that *shrb* was required in GSCs to regulate non-autonomously the number of germ cells per cyst.

### 3. *shrub* loss of function leads to the formation of stem-cysts

**Figure 3:**
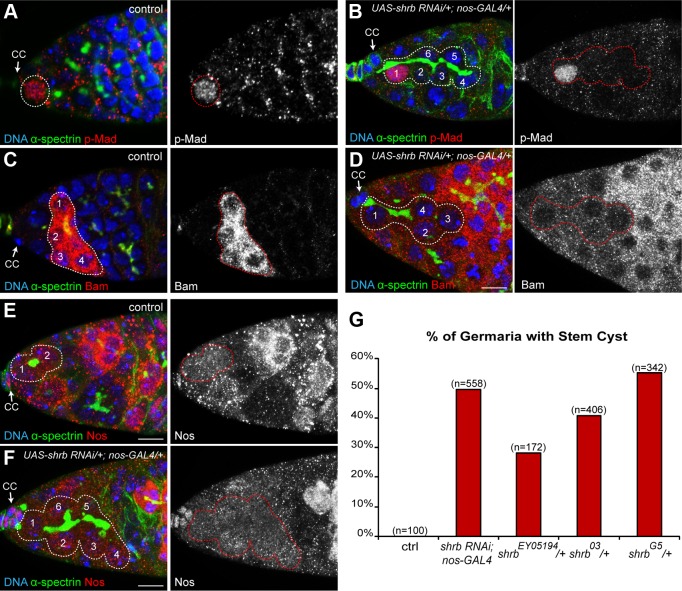
*shrub* loss of function leads to the formation of stem-cysts. Germaria of WT females (A, C, and E) or females expressing *UAS-shrb RNAi* under the control of *nos-GAL4* driver (B, D and F), stained for DAPI (DNA, blue), α-spectrin (fusome, green), and either p-Mad (A and B, red), Bag of marble (Bam, C and D, red), or Nanos (Nos, E and F, red). Dotted lines surround: GSC (A), stem-cysts (B, D and F), 4-cell cyst (C) and GSC/CB pair (E). Cap cells (CC) are indicated. (G) Fraction of germaria exhibiting at least one stem-cyst on the y axis. Genotypes are on the x axis. Scale bar: 10 μm.

The requirement for Shrb in GSCs rather than in cysts to regulate the number of germ cells strongly indicated that Shrb regulates abscission rather than the number of cyst divisions. We had previously shown that a delayed abscission in GSCs led to the formation of group of cells that remained connected by cytoplasmic bridges. We named these clusters “stem-cysts” as they express stem-cell markers but are linked by a branched fusome as found in germline cysts. We thus looked for such stem-cysts in *nanos*-*GAL4; UAS-shrb-RNAi* ovaries. We found that the fusome of mutant GSCs did not have a round or exclamation point shape as a regular GSC, but was instead branched and apparently passing through several cells (Fig. 3A-B). Next, we examined p-Mad staining, a nuclear marker of GSC identity induced by Dpp signal secreted by the cap cells [31]. We found that the mutant GSC attached to the cap cells was positive for p-Mad, as in wild type, indicating that stem cell identity was not affected upon *shrb* loss of function (Fig. 3A-B). In wild type GSC, Dpp signaling blocks the transcription of *bam*, which is necessary and sufficient for the differentiation of the cyst and therefore, Bam protein is not present in the GSC (Fig. 3C-D) [30]. We found that upon *shrb* loss of function, all the cells that were linked to the anterior most GSC by a fusome were devoid of Bam protein. Finally, Nanos protein is weakly expressed in control GSCs, and is completely lost in early differentiating cysts (Fig. 3E)[32]. Upon *UAS-shrb-RNAi* expression, we found weak expression levels of Nanos in the anterior GSC and in cells connected to it by a branched fusome (Fig. 3F). We also observed clusters of cells with the same characteristics in ovaries heterozygous for *shrb^G5^* and *shrb^O3^* alleles. These groups of cells expressed Nanos but not Bam, like wild type GSCs. Moreover, they were also linked by a branched fusome like germline cysts (S2 Fig.) thus corresponding to our definition of stem-cysts. In addition, we found that the penetrance of stem-cysts formation was high 50% (n = 558) for *nanos-GAL4;UAS-shrb-RNAi*, and 55% (n = 342), 41% (n = 406), 28% (n = 172), for germarium heterozygous for *shrb^G5^*; *shrb^O3^* and *shrb^EY05194^*, respectively. The high penetrance of stem-cysts was consistent with the high number of 32-cell cysts.

### 4. Stem-cysts in *shrub* mutant ovaries are caused by synchronous divisions

**Figure 4:**
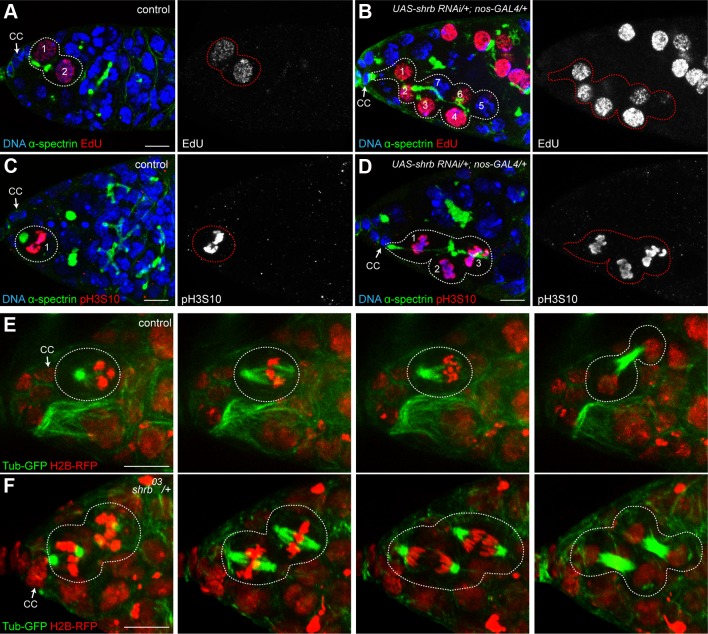
Stem-cysts in *shrub* mutant ovaries are caused by synchronous divisions. Germaria of WT females (A and C) or females expressing *UAS-shrb RNAi* under the control of *nos-GAL4* driver (B and D), stained for DAPI (DNA, blue), α-spectrin (fusome, green), and either EdU (A and B, red) showing S-phase, or pH3S10 (C and D, red) to highlight mitotic cells. Dotted lines surround: GSC/CB pair (A), stem-cysts (B and D) and GSC (C). (E and F) Selected time points of live imaging experiments performed on germaria expressing H2B-RFP (chromatin, red) and G147 (tubulin, green). (E) A WT GSC (surrounded by dotted lines) undergoing mitosis alone. (F) In a female heterozygous for *shrb* (*shrb^03^/+*), the GSC and its daughter CB undergo mitosis synchronously (surrounded by dotted lines). Cap cells (CC) are indicated. Scale bar: 10 μm.

Stem-cysts are formed by the synchronous divisions of GSCs and connected cells, while previous abscissions have not been completed. To test the synchronicity of GSCs with other cells, we first analyzed EdU incorporation after a 15 min pulse to mark the S-phase. In control condition, we found that GSCs and their daughter cystoblasts incorporated similar amount of EdU, indicating that they were still synchronized and connected as previously described [20,22]. However, we never detected additional neighboring cells labelled by EdU (Fig. 4A, n = 45). In germarium expressing *shrb-RNAi*, we observed that in addition to the anterior GSC and its direct neighbor, additional neighboring cells had incorporated EdU, indicating that all these cells had replicated synchronously their DNA (Fig. 4B, n = 9). These cells were all linked by a common fusome. To test if the synchrony of the cell cycle extended to M-phase, we used the mitotic marker pH3. We found that in control conditions, GSCs always divided non-synchronously with their neighbors, including their daughter cystoblasts (Fig. 4C, n = 27). In contrast, in germ cells expressing *shrb-RNAi*, all cells linked to a GSC by a fusome exhibited similar levels of pH3 staining. This indicated that these cells were all performing mitosis synchronously (Fig. 4D, n = 15). Finally, to directly analyze cell cycle synchrony in stem-cysts, we used live imaging of heterozygous *shrb^O3^*/+ ovaries expressing an *H2B-RFP* transgene to label chromosomes, and *G147*, a protein-trap insertion marking microtubules. We were able to follow 10 GSCs undergoing mitosis, and found that 4 of them were dividing synchronously with neighboring cells (Fig. 4F). This result is consistent with the percentage of stem-cysts observed by immunostaining. Synchronous divisions were never observed in wild type control (Fig. 4E, n = 21). Altogether, these results demonstrated that cells within stem-cysts induced by *shrb* loss-of-function were cycling synchronously.

### 5. Cells within one stem-cyst share the same cytoplasm

**Figure 5:**
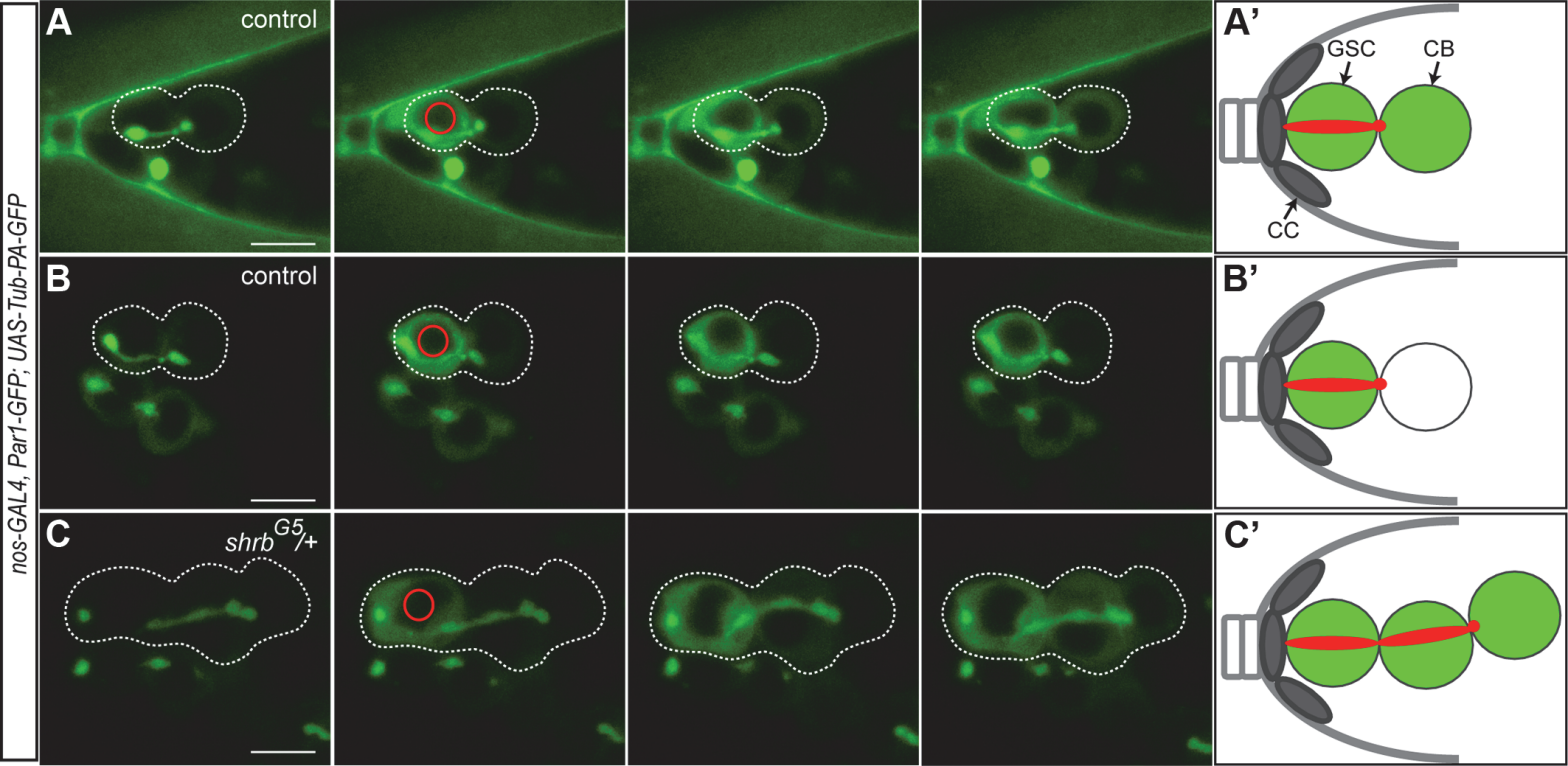
The cells of the stem-cyst share the same cytoplasm. Selected time points of live imaging experiments performed on germaria expressing *UAS-Par1-GFP* and *UAS-Tubulin-PA-GFP* under the *nos-GAL4* promoter. Tub-PA-GFP is photoactivated in the region defined by the red circle in the GSC, and the fluorescence diffusion to the neighboring cells is observed. (A and B) WT female GSCs in mid-G2 phase. (A) Abscission between the GSC and the CB did not yet occurred as the GFP diffuses to the CB. (B) GFP does not diffuse to the CB. Abscission has occurred and the cells no longer share their cytoplasm. (C) Stem-cyst of *shrb^G5^/+* female. After photoactivation in the GSC, the GFP diffuses through the 2 neighboring cells. This shows that the cells of the stem-cyst do not complete abscission and stay connected, sharing their cytoplasm. Dotted lines surround: GSC/CB pair (A and B), stem-cysts of 3 cells (C). (A’, B’ and C’) Schematic representation of the Tubulin-PA-GFP diffusion from the GSC to the CB (A’ and B’) or within a stem-cyst (C’). Scale bar: 10 μm.

The cell cycle synchronicity within stem-cysts suggested that these cells shared the same cytoplasm and that *shrb* loss-of-function induced a strong delay in abscission. To determine precisely when abscission took place in wild type and mutant conditions, we expressed a diffusible α-tubulin tagged with a photo-activatable GFP (Tubulin-PA-GFP). Activation of the GFP in one cell allowed tracing labeled tubulin to the neighboring cell, and thus determining whether or not abscission had been completed. Indirect assays, using EdU incorporation, had already established that abscission between GSCs and cystoblast happens during or after S-phase in wild type condition [20,22]. To time more precisely abscission in GSCs, we used the shape of the fusome as a timer, as it has been shown to follow stereotypical changes during the different phases of the cell cycle [22,33]. We used germaria co-expressing Tubulin-PA-GFP with a *UAS-Par-1-GFP* transgene to label the fusome during live-imaging. We activated Tubulin-PA-GFP in one GSC with a brief pulse of a 2-photon laser, and recorded its diffusion to the attached cystoblast for each stage of the fusome cycle as defined in [33]. We observed diffusion of Tubulin-PA-GFP in the cystoblast only when the GSC and the cystoblast were visibly linked by a fusome (n = 61). We observed diffusion in GSC/CB pairs harboring a fusome shaped as a plug (n = 2, G1 phase), a bar (n = 6, G1/S phase), dumbbell (n = 15, S phase), fusing (n = 16, G2 phase) and an exclamation point (n = 22, G2 phase, Fig. 5A, S1 Movie). However, we also observed some GSC/CB pairs linked by an exclamation point fusome, in which Tubulin-PA-GFP did not diffuse from GSC to CB (n = 21, Fig. 5B, S2 Movie). Tubulin-PA-GFP never diffused when the fusome was round (late G2 phase, n = 13). These results indicate that Tubulin-PA-GFP can diffuse from GSC to CB until the exclamation point stage of the fusome, i.e. mid-G2 phase. We thus established that in wild type condition, abscission between GSC and CB happens during mid-G2 phase (Fig. 5). We carried out the same experiment in *shrb^G5^*/+ mutant GSCs. We selected stem-cysts with at least three cells, including an anterior GSC, linked by a Par1-GFP positive fusome. We activated Tubulin-PA-GFP in the anterior GSC. We observed diffusion of Tubulin-PA-GFP in all cells of a single stem-cyst in 96% stem-cysts analyzed (Fig. 5C, n = 27, S3 Movie). This result demonstrated that cells within one stem cyst shared a common cytoplasm, and that abscission was almost never completed in GSCs, although scission eventually happened at other cytoplasmic bridges of stem-cysts.

### 6. *Drosophila* Shrub localizes on the fusome and at the midbody

**Figure 6:**
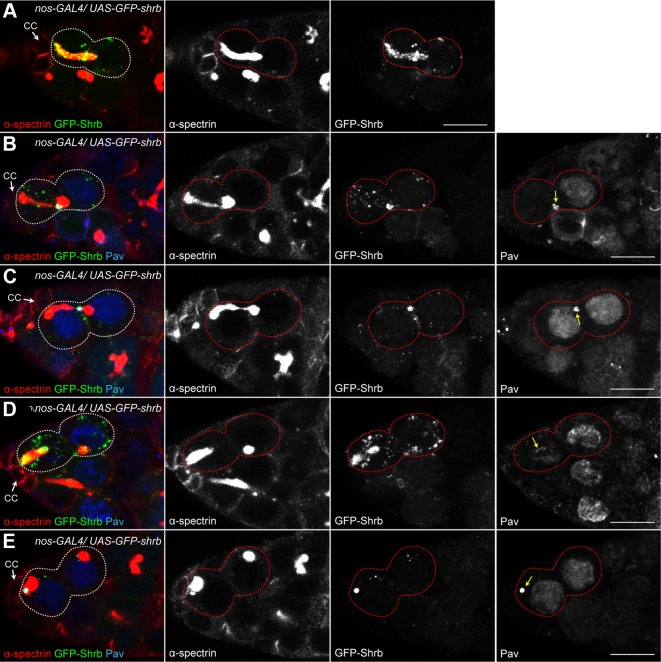
*Drosophila* Shrub localizes on the fusome and at the midbody. Ovaries expressing *UAS-GFP-shrb* under the control of *nos-GAL4* and stained for α-spectrin (A, red) and Pavarotti (B-E, Pav, blue) a marker of the midbody. Midbody position is indicated by a yellow arrow. (A) Before abscission, GFP-Shrb localizes to the fusome (red) and at the ring canal between the GSC and the CB (See also S3B Fig.). (B) GFP-Shrb localizes specifically at the ring canal and midbody (Pav, blue) during its constriction. (C) During abscission, GFP-Shrb is enriched at the midbody, colocalizing with Pav. The ring canal is no longer visible. (D) After abscission, the fusome retracts towards the GSC and the midbody, stained with Pav and Shrb-GFP, is segregated with it. (E) GSCs with round spectrosome (late G2, M) often show colocalization of Shrb-GFP with the midbody (Pav). Scale bar: 10 μm.

To analyze the dynamic localization of Shrb in germ cells, we generated an N-terminal fusion protein, GFP-Shrub (GFP-Shrb), under the control of a *UASp* promoter to express it in GSCs and germline cysts with the *nanos*-GAL4 driver. In GSCs, we found that GFP-Shrb localized as dots along the fusome and at the transient ring canal linking the GSC and CB before abscission (Fig. 6A). In mid-G2 phase, when the fusome adopts an exclamation point shape, we found that GFP-Shrb localized additionally to a strong focus at the site where the fusome splits. We hypothesized that this dot could be the midbody, and therefore co-stained ovaries for the midbody marker Pavarotti (Pav), which is the *Drosophila* homologue of MKLP1. We found a perfect co-localization of GFP-Shrb and Pav on this structure and on the surrounding ring canal (Fig. 6B). This result indicated that GFP-Shrb localized at the midbody. At a later stage, when the fusome between the GSC and the CB is about to break, both Shrb and Pav disappeared from the shrunk ring canal, but remained co-localized at the midbody (Fig. 6C). Similarly, after scission when the fusome is retracting to reform the typically round spectrosome in the GSC, Shrb and Pav co-localized at the posterior tip of the fusome (Fig. 6D). In late G2 and mitosis, we occasionally observed midbodies co-stained by Pav and GFP-Shrb next to the spectrosome (Fig. 6E). This dynamic behavior of GFP-Shrb is consistent with a function of Shrb in GSCs abscission, and with a recent report demonstrating that the midbody is asymmetrically inherited by the GSCs in ovaries [34]. We further observed that this asymmetric inheritance of the midbody occurs concurrently to the retraction of the fusome to reform a round spectrosome. In germline cysts of the mitotic region (region 1), we found that GFP-Shrb was almost not visible even in cytoplasmic vesicles (S3 Fig.). In contrast, in meiotic cysts (region 2 of the germarium), GFP-Shrb localized as bright dots along the fusome and at ring canals (S3 Fig.).

While studying the localization of GFP-Shrb, we noticed a weak but consistent appearance of 32-cell cysts in an otherwise wild type background. Depending on the expression levels of different insertions of *UASp-GFP-Shrb* transgenes, and using the same *nanos*-GAL4 driver, we observed between 4% and 15% of egg chambers with 32 cells. This result suggested that expression of GFP-Shrb could have a dominant-negative effect, as it gave the same phenotype as a loss-of-function of *shrub*. Alternatively, a high expression of Shrb could induce the same phenotype as a low expression. To distinguish between these alternatives, we expressed GFP-Shrb in a *shrb* heterozygous background. If GFP-Shrb acts as a dominant-negative allele, it should aggravate the 32-cell phenotype, while it should rescue it if GFP-Shrb is a functional protein. We found that the phenotype of 32-cell cysts decreased from 44% in *shrb^O3^*/+ ovaries to 12% in *shrb^O3^*/+ ovaries expressing GFP-Shrb (S3 Fig.). We concluded that GFP-Shrb was not a dominant-negative allele, and that abscission was very sensitive to the levels of Shrb whether increased or reduced.

### 7. Loss of Lethal giant discs (Lgd) induces the formation of stem-cysts and 32-cell egg chambers

**Figure 7:**
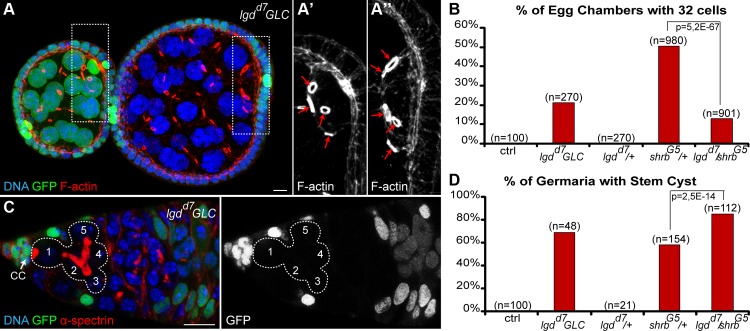
Loss of Lethal giant discs induces the formation of stem-cysts and 32-cell egg chambers. (A) Female germline clones (GLC) of *lgd^d7^* (*hsFlp/+; lgd^d7^, FRT 40A/ GFP, FRT 40A*) stained with DAPI (DNA, blue) and phalloidin (F-actin, red). Egg chamber with heterozygous germ cells (GFP, green) is made of 16 cells with a 4 ring canals oocyte (close up in A’). The neighboring egg chamber is a GLC, made of 32 cells with a 5 ring canals oocyte (close up in A’’). (B) Fraction of egg chambers exhibiting 32 cells on the y axis. Genotypes are on the x axis. (C) Female germline clones (GLC) of *lgd^d7^* stained for DAPI (DNA, blue) and α-spectrin (fusome, red). A stem-cyst composed of 5 homozygous mutant cells is shown. (D) Fraction of germaria exhibiting at least one stem-cyst on the y axis. Genotypes are on the x axis. Scale bar: 10 μm.

Lgd is a tumor suppressor known to regulate endosomal trafficking and is a direct interactor of Shrb. In *Drosophila* and vertebrates, Lgd was shown to interact physically with Shrb via its DM14 domain. This interaction is required in flies for Shrub endosomal function [35]. In contrast, the mammalian homologue of Lgd was suggested to be an inhibitor of CHMP4B, a Shrb orthologue [36]. To assess the function of Lgd in germ cell abscission, we induced germline clones homozygous mutant for null alleles of Lgd. We found that 21% (n = 270) of *lgd^d7^* mutant egg chambers contained 32 cells (Fig. 7A-B). Furthermore, we observed that 69% of *lgd^d7^* mutant GSC formed stem-cysts (Fig. 7C-D, n = 48). We concluded that in the absence of Lgd, abscission is delayed in GSCs, and thus that Lgd is required positively for abscission to be completed. To investigate whether Lgd and Shrb act in the same pathway to regulate abscission, we performed genetic interactions between *lgd* and *shrb* alleles by crossing heterozygous *shrb^G5^*/+ flies with *lgd^d7^* null allele. We found that the number of stem-cysts was significantly increased in *shrb^G5^*/*lgd^d7^* (85%, n = 112) compared to *shrb^G5^*/+ (58%, n = 154) (Fig. 7D). We concluded that Lgd and Shrb interacted positively for GSCs abscission. Surprisingly, we found that the number of egg chambers with 32 cells was reduced in double heterozygous flies. We counted 13% (n = 901) of egg chambers with 32 cells in *shrb^G5^*/ *lgd^d7^* flies, compared to 51% (n = 980) in *shrb^G5^*/+ flies (Fig. 7B). This negative genetic interaction between *lgd* and *shrb* suggested that Lgd might have another function in germline cysts. We concluded that Lgd and Shrb interacted positively in GSCs and negatively in germline cysts.

### 8. Aurora-B and Shrub interact to regulate abscission in germline stem cells

**Figure 8:**
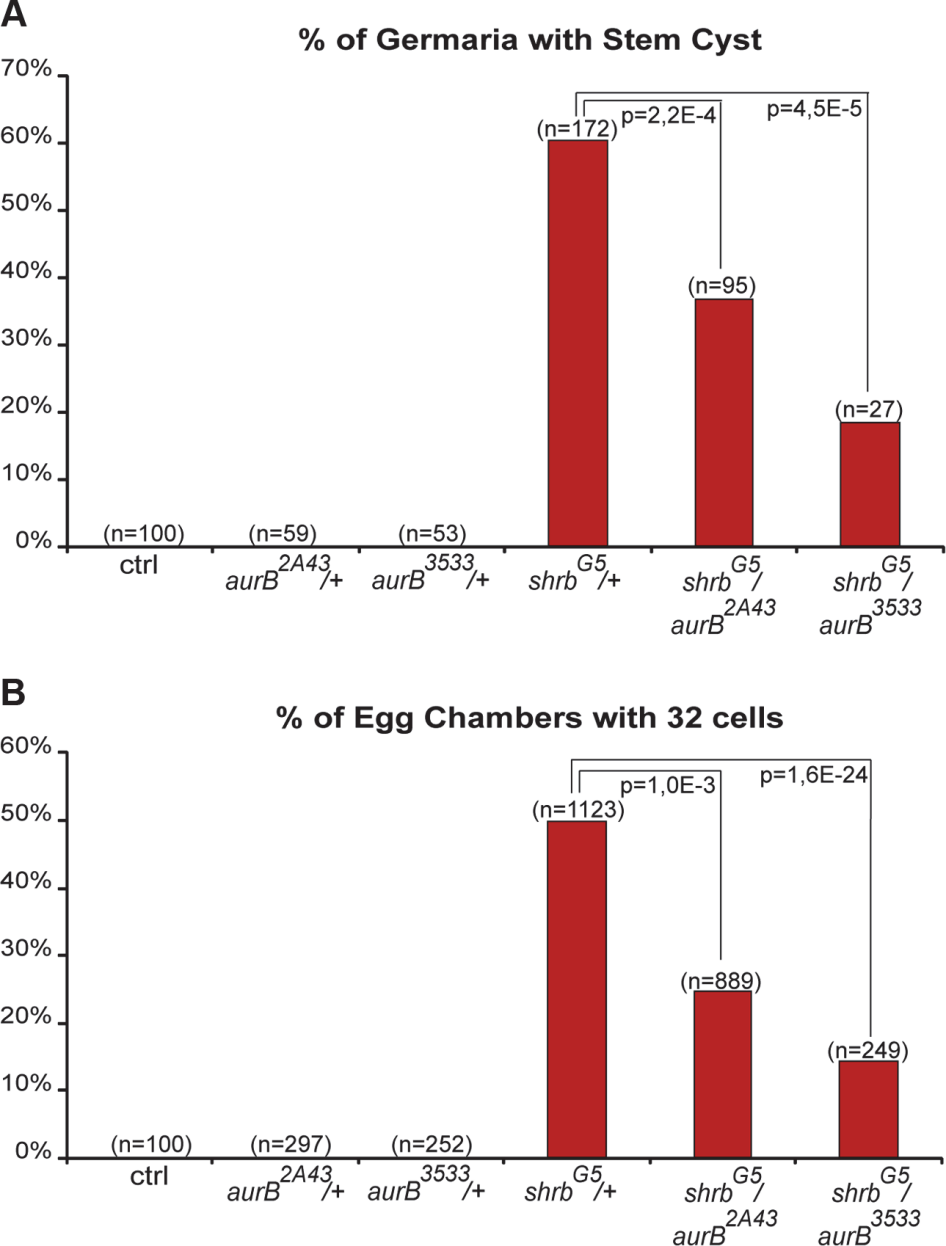
Aurora-B and Shrub interact to regulate abscission in germline stem cells. (A) Fraction of egg chambers exhibiting 32 cells on the y axis. Genotypes are on the x axis. (B) Fraction of germaria exhibiting at least one stem-cyst on the y axis. Genotypes are on the x axis.

In human cells, CHMP4A, B and C are three isoforms of CHMP4, the vertebrate homologue of Shrb. Both CHMP4B and C regulate abscission timing in vertebrate cells, albeit with opposite activity. CHMP4B is known to regulate positively abscission, whereas CHMP4C can delay it [17,37]. The activity of CHMP4C is regulated by Aurora B (AurB)-dependent phosphorylation [17,18]. We previously showed that in flies, AurB negatively regulates abscission, and that abscission occurs precociously in GSCs and 2-cell cysts mutant for *aurB*. In contrast, increasing the activity of AurB leads to the formation of stem-cysts as observed in *shrub* loss-of-function [20]. Shrub was further shown to interact physically with Borealin, a regulatory subunit of the AurB complex [18]. We thus tested genetic interactions between *shrb^G5^*/+ flies and two null alleles of *aurB, aurB^2A43^* and *aurB^3533^*. Both single alleles *aurB^2A43^* and *aurB^3533^* have no phenotype when heterozygous (Fig. 8A-B). We found that the number of stem-cysts in double heterozygous flies *shrb^G5^*/*aurB^2A43^* (37%, n = 95) and *shrb^G5^*/*aurB^3533^* (19%, n = 27) was greatly reduced compared to *shrb^G5^*/+ flies (60%, n = 172) (Fig. 8A). Furthermore, the number of egg chambers with 32 cells was also rescued from 50% (n = 1123) in *shrb^G5^*/+ flies to 25% (n = 889) in *shrb^G5^*/*aurB^2A43^*, and 14% (n = 249) in *shrb^G5^*/*aurB^3533^* ovaries (Fig. 8B). These results demonstrated that AurB negatively interacts with Shrb during GSCs and germline cysts divisions.

## Discussion

The duration of abscission is developmentally regulated in different cell types. We have previously shown that negative feedback loops between two kinases, AurB and CycB/Cdk-1, control the timing of abscission in *Drosophila* germline cells and in vertebrate cells in culture [20]. This mechanism is thus probably conserved in many different cellular contexts. In a genetic screen for genes regulating the number of germ cells per egg chamber, we found that loss of Shrb induced the same phenotype as the overactivation of AurB. Shrb seemed an interesting candidate as it is the *Drosophila* homologue of yeast SNF7 and mammalian CHMP4B/CHMP4C, which have been previously implicated in the regulation of abscission [17]. Furthermore, *Drosophila* Shrb was shown to physically interact with Borealin, a regulatory subunit of the AurB complex [18]. Interestingly, CHMP4B and CHMP4C have opposite effect on abscission; CHMP4B is a positive regulator of abscission, while CHMP4C delays abscission when phosphorylated by AurB [17,37]. Our results demonstrate that Shrb is a positive regulator of abscission in GSCs, as reducing Shrb levels delayed abscission and led to the formation of stem-cysts. In addition, we found that reducing AurB levels could rescue a reduction of Shrb levels for the formation of both stem cysts and 32-cell egg chambers. This negative genetic interaction indicates that a delay in abscission caused by lower levels of Shrb can be compensated by lower levels of AurB, which accelerate abscission. We also analyzed genetic interactions between *shrb* and *lgd*, as Lgd is known to be required for Shrub function in *Drosophila* [35]. Accordingly, we found that loss of *lgd* induced the formation of stem-cysts and 32-cell egg chambers. Furthermore, decreasing Lgd levels in a *shrb* mutant background increased the delay in abscission as shown by the higher number of stem-cysts. However, we observed surprisingly that the 32-cell phenotype was rescued in transheterozygous *shrb^G5^*/*lgd^d7^* females. This result indicated that *lgd* and *shrb* interacted negatively in the cyst, contrasting with the positive interaction they showed in the GSC for abscission. We propose that *lgd* may have another function in the cyst that can compensate for *shrb* loss of function. We speculate that Shrub is required for the maintenance of the incomplete cytokinesis of the cyst cells. Reduced levels in *shrb^G5^*/ *lgd^d7^* females would allow the presumptive 32-cell cysts originating from 2-cell precursors to break into two 16-cell cysts, as observed in *aurB* loss-of-function. The “rescued” 16-cell cysts could thus result from a combination of two successive phenotypes, delayed abscission in GSCs followed by a failure to maintain incomplete abscission in cysts at the oldest ring canal. Unfortunately, it is not currently possible to remove *lgd* function only in germline cysts to test this hypothesis. In addition, Lgd and Shrb are both involved in endosomal sorting, and their loss of function may therefore affect signaling pathways and other processes, complicating the interpretation of genetic interactions [38,39,40,41]. Indeed, we observed that loss of Shrb also induced additional phenotypes in ovaries, such as tumorous egg chambers.

One remarkable finding of our study is the great sensitivity of abscission to the levels of Shrb. We observed a gradation in the penetrance of phenotypes depending on the levels of Shrb in the germline. A moderate overexpression of GFP-Shrb using the *nanos*-GAL4 promoter produced less than 15% of egg chamber with 32 cells. Removing one copy of *shrb* induced up to 60% of 32-cell cysts, and the appearance of polyploid germ cells in the germarium. These polyploid cells probably resulted from a complete failure of cytokinesis, and we interpret it as a stronger phenotype than the formation of stem-cysts. Stronger phenotypes were obtained by using shRNAs targeting *shrub*, with up to 80% of egg chambers with 32 cells, many polyploid cells and in addition, the formation of egg chambers containing tumor-like germ cells. Finally, we could not even obtained homozygous *shrb* mutant cells using the Flp/FRT technique indicating that Shrb is required for cell viability in the germline. The levels of Shrb thus appear to be essential for its proper functions, including the regulation of abscission timing in GSCs. In mammalian cells, the final step of abscission is thought to be mediated by the formation of 17 nm-diameter filaments spiraling from the midbody to the constriction zone. The formation of these filaments depends on the Shrb homologue, CHMP4B, and helices of CHMP4B have been described by structured illumination microscopy [6]. However, even though CHMP4B can form filaments in cells, the filaments formed in these CHMP4B over-expressing cells had only a diameter of 5 to 6 nm[42]. Therefore, it is tempting to speculate that the helical filaments observed during abscission are hetero-polymers, comprising other components in addition to Shrb/CHMP4B subunits. Changing the stoichiometry of these components by decreasing or elevating the levels of Shrb/CHMP4B may affect the ability of the subunits to form filaments of the proper diameter to perform abscission. This could explain why overexpression of Shrb or removing one copy of *shrb* can both lead to a delay in abscission.

In mammalian cells, delay in abscission often induces the regression of the cytoplasmic furrow and the formation of bi-nucleated cells [16]. In contrast, we found that in *Drosophila* germ cells, abscission delay resulted in the formation of stem-cysts. In stem-cysts, we demonstrated that all cells shared the same cytoplasm as shown by the diffusion of Tubulin-GFP between ring canals. However, each cells remained individualized and only the most anterior cell, i.e. the cell in contact with the niche, had pMad translocated in the nucleus. This result could be explained if pMad cannot diffuse in the cytoplasm. This intriguing result could also be in agreement with the proposal that cells away from the niche are actively induced to differentiate by neighboring escort cells [43]. We speculate that posterior escort cells can promote the differentiation of distal cells in stem-cysts. Stem-cysts are formed by several rounds of mitosis of GSCs before the completion of preceding abscissions. These mitoses are synchronous and thus form stem-cysts of 2, 4 or 8 cells. However, abscission is only delayed, and ultimately takes place at the oldest ring canals, releasing cystoblasts made of 2 or 3 cells. These multicellular cystoblasts then undergo the regular four mitosis giving rise to 32-cells cyst (2×16) originating from 2-cell cystoblasts; or 48-cell cysts (3×16) originating from 3-cell cystoblasts. In this study, we have only occasionally observed 48-cell cysts, obtained with a strong over-activation of AurB. Reduction of *shrb* or *lgd* mostly generated 32-cell cysts, originating from 2-cell CBs. In agreement, we found stem-cysts mainly formed of 4 cells. We can thus speculate that abscission takes about twice as long in these mutant conditions.

In mammalian cells, the recruitment of ESCRT proteins to the midbody is tightly regulated in time to prevent premature abscission [1]. Intriguingly, we found that in *Drosophila* GSCs, GFP-Shrb localizes on the fusome long before abscission, and also later on at the midbody, which remains associated with the fusome. Since Shrub is present at the site of scission before abscission takes place, its activity must be inhibited to prevent premature abscission. Our genetic interactions between *shrb* and *aurB* suggest that AurB and the CPC could inhibit Shrb activity. There is still no evidence in *Drosophila* that AurB phosphorylates Shrb, as the residues phosphorylated by AurB in CHMP4C are not conserved in *Drosophila* Shrub. In contrast, it has been proposed that direct binding between Shrub and Borealin, a member of the AurB—CPC complex, could block Shrb activity by keeping Shrb in a closed conformation [18]. Consistent with this hypothesis, we have shown that Survivin, another member of AurB complex, is localized on the fusome in GSCs [20]. It is thus possible that precocious activity of Shrb in GSCs is prevented by its binding to the CPC on the fusome. Interestingly, we could barely detect GFP-Shrb on the fusome in dividing cysts when abscission remains incomplete. This indicates that Shrb levels are regulated developmentally, and that there is a correlation between the absence of Shrub protein on the fusome and incomplete cytokinesis in germline cysts. Furthermore, GFP-Shrub was expressed using the exogenous *nanos*-Gal4 promoter (i.e. not under the endogenous transcriptional regulation) indicating that Shrub is regulated at the protein level. This raises the exciting possibility that the absence of Shrb in dividing cysts may block abscission in differentiating germline cysts. Elegant works performed in the mouse testis have shown that TEX14 blocks abscission in spermatogonia [14]. There is no homologue of TEX14 in *Drosophila*, and what blocks abscission in the differentiating cysts remains a major question in the field. We believe that elucidating how Shrb protein levels are regulated in the fly germline cyst may help understand how incomplete cytokinesis is controlled.

## Materials and Methods

### *Drosophila* genetics

The *Drosophila* alleles or transgenes used in this study are HMS01767 (TRiP line; [29]); *shrb^G5^* and *shrb^03^* [41]; *shrb^EY05149^* (Bloomington Stock Center); *H2B-RFP* [44]; *G147* [45]; *UASp-tubulin-PA-GFP* [46]; *lgd^d7^* [47]; *lgd^EY04750^* (Bloomington Stock Center); *aurB^2A43^* and *aurB^3533^* [20]; *UASp-par1-GFP*[48].

Overexpression experiments were performed using the Gal4/UASp system [49] with the *nanos-GAL4-VP16* [50] or the *bam-GAL4* [51] drivers. We generated the *bam*-GAL80 construct (see later), and combined it with a *nanos*-GAL4 driver devoid of VP16 activation domain (a kind gift of M. Fuller).

The germline clones were generated using the Flp/FRT technique [52,53]. Clones were induced by heat-shocking third instar larvae at 37°C for 2 hours, females were dissected 2 days after eclosion.

### Constructs

To generate the *bam*-Gal80 construct, we synthetized a 3886 bp DNA fragment encoding the *bam* promoter (−898 to +133, between 5’ AGATCTAACCATTGATTAAC 3’ and 5’GATTTGTGTGATTTAACTTA 3’), the Gal80 coding sequence (5’ ATGGACTACAACAAGAGAT 3’ to 5’ TCTCGCATTATAGTTTATAA 3’) and terminated by the K10 terminator, between Not1 sites (eurofins/MWG). The NotI fragment was then cloned into pCasper vector.

To generate the pUASp-GFP-shrb construct, we subcloned the shrb fragment from a pDONR221 vector for Nter fusion construct (a kind gift from Pier Paolo D’Avino; [18]) by LR recombination to pPGW destination vector.

Transgenic lines were generated by BestGene.

### Immunohistochemistry

Antibody staining and Hoechst staining were performed according to standard protocols. Briefly, ovaries were dissected in PBS, fixed in 4% PFA, permeabilized in PBT (PBS-0,2%Triton) for 30 min, left overnight with primary antibodies in PBT at 4°C, washed 2 h in PBT, left with secondary antibody for 2 hrs at room temperature, washed 1 h in PBT and mounted in Cityfluor.

The primary antibodies used in flies were the following: mouse-anti-α-spectrin (clone 3A9, DSHB) 1:500; rb anti-α-spectrin 1:1000 [54], rat-anti-BamC 1:1000 [30]; rb-anti-Nanos 1:200 [55]; rb-anti-pSMAD 1:100 (a king gift of Peter ten Dijke); rb-anti-Pav 1:150 [56], rb-anti pH3S10 1:1000 (Upstate). Fluorescent secondary antibodies were from Jackson Immunoresearch; rhodamin-phalloïdin and Hoechst were from Molecular Probes.

For Edu treatment (Click-iT Edu Imaging kit, Invitrogen), ovaries were dissected in Schneider medium complemented with 10% FBS. There were then incubated at 25°C for 15 min in 20μM Edu in Schneider medium+ 10% FBS. Edu detection was performed according to manufacturer’s instructions.

### Quantification and statistics

The number of cell per egg chamber was quantified on DAPI and Rhodamin-phalloïdin stained ovaries. We counted the number of nuclei with the DAPI staining. In addition, the number of ring canals stained by phalloïdin was counted for the oocyte so that chambers formed of 32 cells having 2 oocytes with 4 ring canals each (due to encapsulation defects) are not included. Quantification of the percentage of egg chambers having more or less than 16 cells were done on one day old females. Chi-square tests were used to compare the proportions of egg chambers having 16 or 32 cells.

Stem-cysts quantification was done on germaria immunostained with alpha-spectrin antibody; stem-cysts were identified as a group of 3 cells minimum, linked by a fusome, with its most anterior cell being attached to the niche. The percentage of germaria exhibiting at least one stem-cyst was counted. Chi-square tests were used to compare the percentages observed in the different genotypes.

### Microscopy

Acquisition of Z-stacks on fixed sample was carried out on Zeiss LSM710 or LSM780 confocal microscopes. For quantification of egg chambers with 16 or 32 cells, ovaries were analyzed with a Upright Widefield Leica Microscope.

For live imaging of germarium in Fig. 4, ovaries were dissected and mounted in oil (10S, Halocarbon, Sigma) and were imaged with an inverted Confocal Spinning Disk Roper/Nikon equipped with a CCD camera CoolSnap HQ2. Time-lapse images were then treated with Fiji.

For the photo-activation experiments of Fig. 5, ovaries were dissected and mounted in oil (10S, Halocarbon, Sigma). Photo-activation was done with a 2-photon laser at 820nm (3 iterations, laser power 15%, scan speed = 6; these numbers are rough approximations for the excitation power). Imaging was done with a confocal microscope Zeiss LSM 710.

## Supporting Information

S1 Fig Description of the time course phenotypes of *shrub* downregulation. Females expressing *UAS-shrb RNAi* under the control of *nos-GAL4* were dissected after 1, 2, 3 or 5 days. (A) Phenotypes in the cyst: apart from the WT looking chamber of 16 cells and those with 32 cells, chambers with encapsulation defects (B and C), as well as “tumor bags” (D) were observed. (E) Phenotypes in the germarium: apart from the WT looking cells, cells highly polyploid (F, yellow * indicates a polyploidy cell, white * indicates normal cell) and germaria without germ cells (G, stem cell loss) were observed. (B, C, D and G) Ovaries were stained for DAPI (DNA, blue) and phalloidin (F-actin, red). (F) Ovaries were stained for DAPI (DNA, blue) and α-spectrin (fusome, red). Scale bar: 10 μm.
(TIF)

S2 Fig Stem cyst in *shrub* heterozygous. Germaria of WT females (A and C) or *shrb^03^/+* females (B and D), stained for DAPI (DNA, blue), α-spectrin (fusome, green), and either Nanos (A and B, red), or pH3S10 (C and D, red) to highlight mitotic cells. Dotted lines surround: GSC/CB pair (A), stem-cysts (B and D) and GSC (C). Scale bar: 10 μm.
(TIF)

S3 Fig Over-expression of GFP-Shrub. (A) Ovaries expressing *UAS-GFP-shrb* under the control of *bam-GAL4* were stained for DAPI (DNA), α-spectrin (red). GFP-Shrb (green) is expressed only in the early (mitotic) cysts in the germarium, at low levels. (B) Ovaries expressing *UAS-GFP-shrb* under the control of *nos-GAL4* were stained for DAPI (DNA), α-spectrin (red). GFP-Shrb (green) is present in all germline. In the GSC and CB it is enriched in the fusome, ring canal (check also Fig. 6) and vesicles; it stays present also in the 2 cell cyst (cc), but it barely detected in 4, 8 and 16cc; in meiotic 16cc, GFP-Shrb localizes to vesicles enriched at the fusome. (C) Fraction of egg chambers exhibiting 32 cells on the y axis. Genotypes are on the x axis. (D) Females expressing *UAS-GFP-shrb* under the control of *nos-GAL4* were stained for DAPI (DNA), α-spectrin (red); stem-cysts were observed. Scale bar: 10 μm.
(TIF)

S1 Movie Diffusion of Tubulin-PA-GFP from the GSC to its daughter CB in late G2 wild type GSC/CB pair. Wild type GSC/CB pair expressing *UAS-PAR1-GFP* (fusome) and *UAS-Tubulin-PA-GFP* under the control of *nos-GAL4* driver. The GSC and the CB are linked by an exclamation point shaped fusome. Tubulin-PA-GFP was activated in the GSC cytoplasm between the first and second time points. Note the diffusion of the Tubulin-PA-GFP from the GSC to the CB. Time frame: 10 sec
(AVI)

S2 Movie No diffusion of Tubulin-PA-GFP from the GSC to its daughter CB in late G2 wild type GSC/CB pair. Wild type GSC/CB pair expressing *UAS-PAR1-GFP* (fusome) and *UAS-Tubulin-PA-GFP* under the control of *nos-GAL4* driver. The GSC and the CB are linked by an exclamation point shaped fusome. Tubulin-PA-GFP was activated in the GSC cytoplasm between the first and second time points. Note the absence of diffusion of the Tubulin-PA-GFP from the GSC to the CB. Time frame: 10 sec
(AVI)

S3 Movie Diffusion of Tubulin-PA-GFP from the anterior GSC to all cells of the stem cyst in *shrb^G5^*/+ female. Stem cyst of three cells expressing *UAS-PAR1-GFP* (fusome) and *UAS-Tubulin-PA-GFP* under the control of *nos-GAL4* driver in *shrb^G5^*/+ female. The GSC is linked by a linear fusome to two cells. Tubulin-PA-GFP was activated in the GSC cytoplasm between the first and second time points. Note the gradual diffusion of the Tubulin-PA-GFP from the anterior GSC to more posterior cells of the stem cyst. Time frame: 10 sec.
(AVI)
