# Supplementary figures and images for: Abscission Is Regulated by the ESCRT-III Protein Shrub in Drosophila Germline Stem Cells
Source: PLoS Genet. 2015 Feb 3;11(2):e1004653. doi: 10.1371/journal.pgen.1004653 (PMC4372032; doi:10.1371/journal.pgen.1004653)

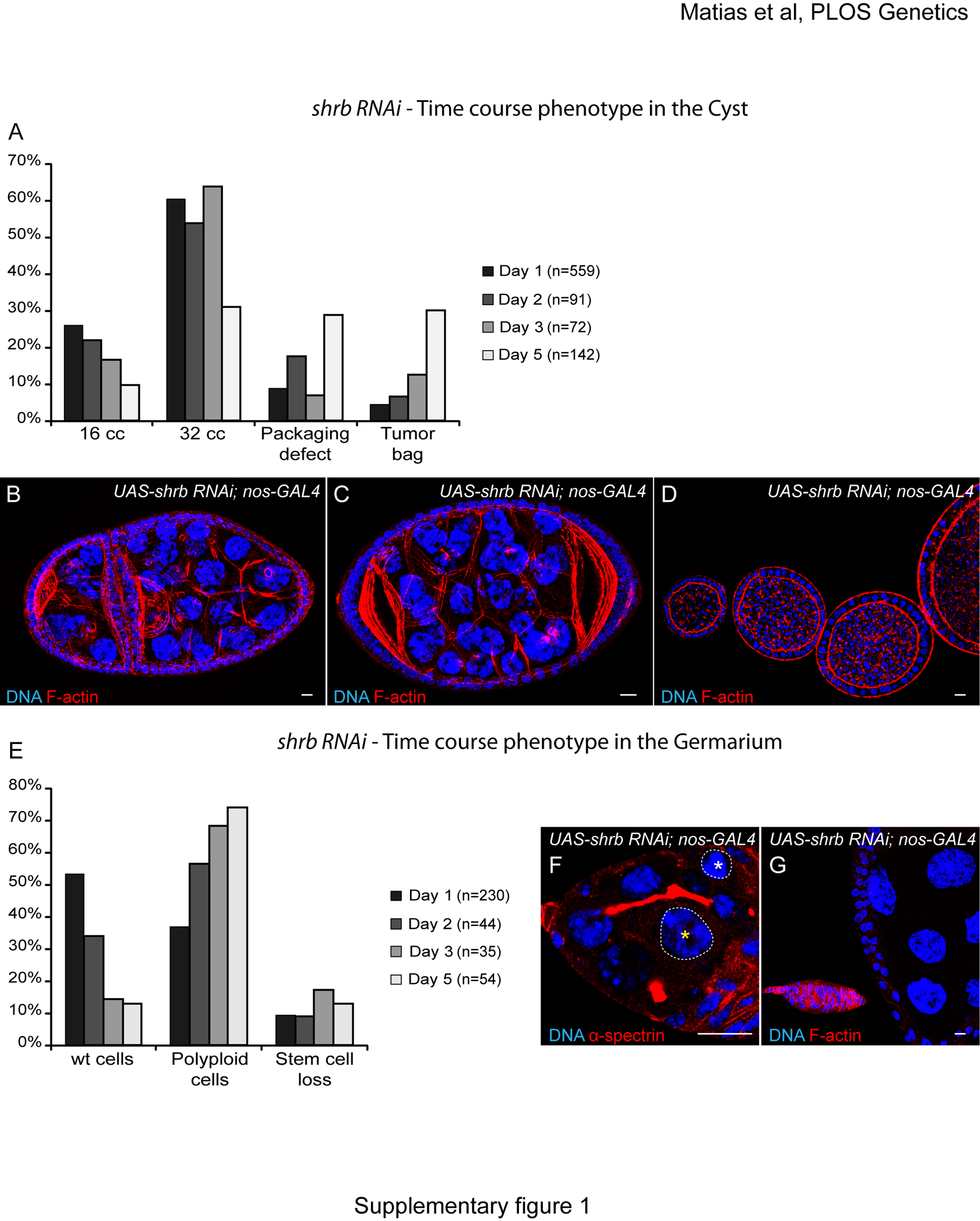

Supplement: S1 Fig — Females expressing UAS-shrb RNAi under the control of nos-GAL4 were dissected after 1, 2, 3 or 5 days. (A) Phenotypes in the cyst: apart from the WT looking chamber of 16 cells and those with 32 cells, chambers with encapsulation defects (B and C), as well as “tumor bags” (D) were observed. (E) Phenotypes in the germarium: apart from the WT looking cells, cells highly polyploid (F, yellow * indicates a polyploidy cell, white * indicates normal cell) and germaria without germ cells (G, stem cell loss) were observed. (B, C, D and G) Ovaries were stained for DAPI (DNA, blue) and phalloidin (F-actin, red). (F) Ovaries were stained for DAPI (DNA, blue) and α-spectrin (fusome, red). Scale bar: 10 μm. (TIF) [file pgen.1004653.s001.tif]

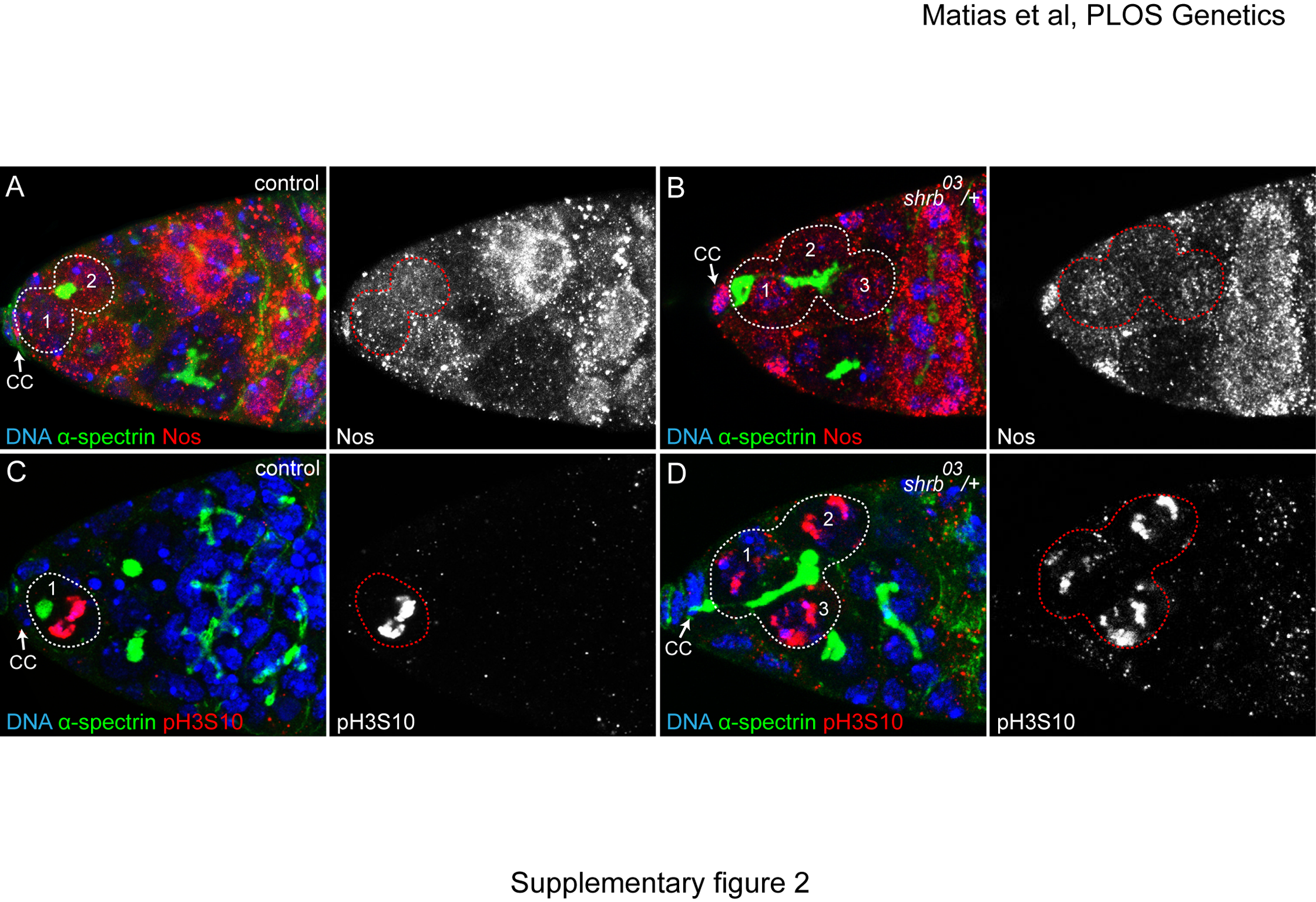

Supplement: S2 Fig — Germaria of WT females (A and C) or shrb03/+ females (B and D), stained for DAPI (DNA, blue), α-spectrin (fusome, green), and either Nanos (A and B, red), or pH3S10 (C and D, red) to highlight mitotic cells. Dotted lines surround: GSC/CB pair (A), stem-cysts (B and D) and GSC (C). Scale bar: 10 μm. (TIF) [file pgen.1004653.s002.tif]

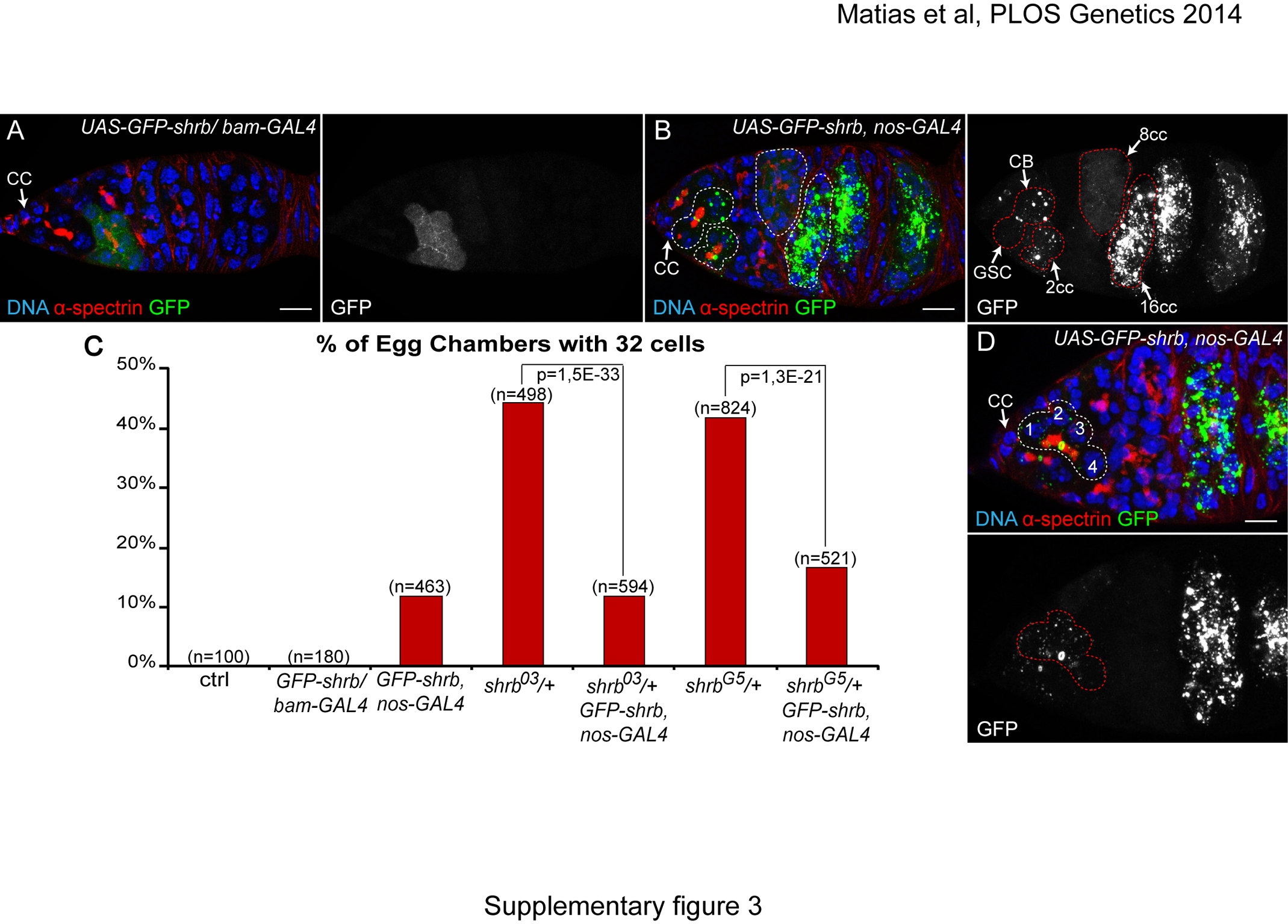

Supplement: S3 Fig — (A) Ovaries expressing UAS-GFP-shrb under the control of bam-GAL4 were stained for DAPI (DNA), α-spectrin (red). GFP-Shrb (green) is expressed only in the early (mitotic) cysts in the germarium, at low levels. (B) Ovaries expressing UAS-GFP-shrb under the control of nos-GAL4 were stained for DAPI (DNA), α-spectrin (red). GFP-Shrb (green) is present in all germline. In the GSC and CB it is enriched in the fusome, ring canal (check also Fig. 6) and vesicles; it stays present also in the 2 cell cyst (cc), but it barely detected in 4, 8 and 16cc; in meiotic 16cc, GFP-Shrb localizes to vesicles enriched at the fusome. (C) Fraction of egg chambers exhibiting 32 cells on the y axis. Genotypes are on the x axis. (D) Females expressing UAS-GFP-shrb under the control of nos-GAL4 were stained for DAPI (DNA), α-spectrin (red); stem-cysts were observed. Scale bar: 10 μm. (TIF) [file pgen.1004653.s003.tif]
